# A rare association between multiple sclerosis and Charcot‐Marie‐Tooth type 1B

**DOI:** 10.1002/brb3.580

**Published:** 2016-09-25

**Authors:** Rosa Cortese, Stefano Zoccolella, Maria Muglia, Alessandra Patitucci, Antonio Scarafino, Damiano Paolicelli, Isabella Laura Simone

**Affiliations:** ^1^ Department of Basic Medical Sciences, Neurosciences and Sense OrgansUniversity of BariBariItaly; ^2^ Institute of Neurological SciencesNational Research CouncilMangone, CosenzaItaly

## Abstract

The association between multiple sclerosis (MS) and hereditary and sporadic demyelinating disorders of the peripheral nervous system is extremely rare. We herein report a case of Charcot‐Marie‐Tooth disease type 1B with p.Val102fs mutation in the MPZ gene that developed relapsing remitting MS.

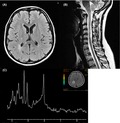

The association between multiple sclerosis (MS) and hereditary (Kawamura et al., 2013) and sporadic (Potulska‐Chromik et al., 2012) demyelinating disorders of the peripheral nervous system is extremely rare. Nevertheless, the contemporary involvement of both central and peripheral myelin could be related to a unique autoimmune pathogenetic mechanisms allowed by the partial homology among peripheral and central proteins (Kawamura et al., 2013; Potulska‐Chromik et al., 2012).

Herein we describe a case of Charcot‐Marie‐Tooth disease type 1B (CMT1B) who developed relapsing MS.

A 40‐years‐old woman was admitted to our hospital complaining of a 10 years history of bilateral progressive weakness and numbness of the lower limbs, followed by loss of strength and difficulty with fine motor skills from a few months before the observation.

She also referred a history of bulimic alimentary disturbance when she was 34, fully recovered after 3‐year treatment with paroxetine (20 mg/daily). Because of this disorder the patient underwent a brain MRI that showed two T2 hyperintense, nongadolinium‐enhancing lesions of right semiovale center and corona radiata, initially interpreted as gliosis.

On admission, the neurological examination revealed ataxic gait, impossible on toes and heels, horizontal exhaustible nystagmus, mild distal muscle weakness (MRC = 4) of the four limbs more prominent on the right side, where the deep tendon reflexes were increased, with Hoffman and Babinski sign. There was also a moderate reduction in the vibration sensation in the four limbs, bilateral pes cavus, and urinary incontinence. Disability score on Expanded Disability Status Scale (EDSS) was 4.5. Standard blood test revealed a mild increase in antinuclear (ANA 1/80) and anti‐*Saccharomyces cerevisiae* (ASCA 22.1 pg/ml; normal: 0–10) antibodies, and a mild reduction in D‐vitamin level (14.9 ng/ml; normal: 30–100). CK level was normal and antigangliosides, NMO, and aquaporin 4 antibodies were absent.

Brain MRI showed several new T2‐hyperintense lesions involving frontal, parietal, and temporoparietal white matter, corpus callosus, and semioval centers, with the right parietal lesion showing gadolinium enhancement (Fig. 1A). Additionally, spinal cord MRI revealed right‐side C4 and C5 lesions with no contrast enhancement (Fig. 1B). Proton (1H) MR spectroscopy with volumes of interest localized on the right posterior parietal region lesion displayed a decrease in N‐acetylaspartate (NAA) and an increase in choline (Cho; Fig. 1C).

**Figure 1:**
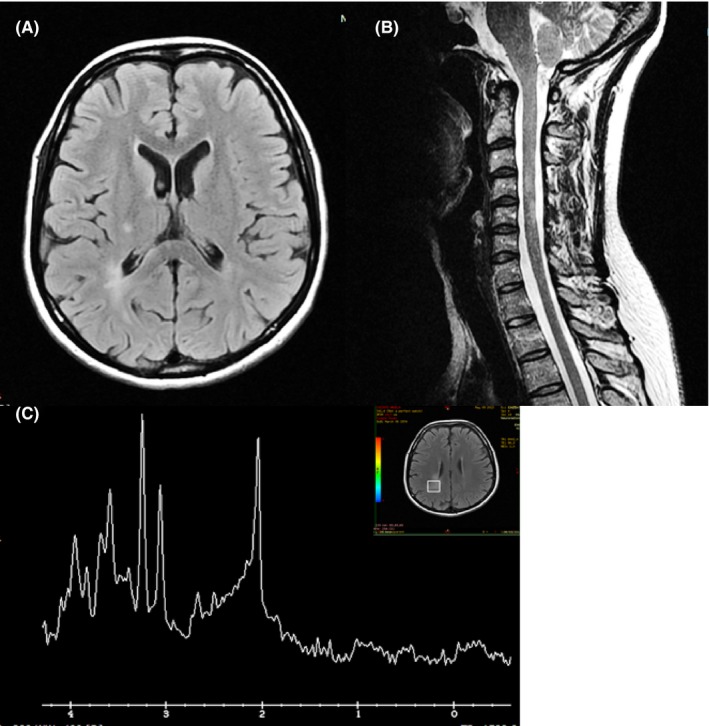
MR FLAIR images showing hyperintense lesions in the right parietal region (A) and in the cervical spinal cord at C4 and C5 (B). Spectroscopy at TE 144 ms displaying high‐NAA and low‐Cho peaks (C)

Sensory evoked potentials showed a delay in both peripheral and central responses in the four limbs, while visual and brainstem potentials were normal. Nerve conduction studies showed a diffuse reduction in both sensory (mean conduction velocity: 36 m/s in lower limbs and 42 m/s in the upper limbs nerves) and motor conduction (mean conduction velocity: 25 m/s in lower limbs and 35 m/s in the upper limbs nerves), with secondary axonal damage. Routine cerebrospinal fluid analysis was normal, but nine oligoclonal bands were found at immunoblotting. A genetic study of the genes responsible for Charcot‐Marie‐Tooth showed in heterozygous state the mutation p.Val 102fs in the exon 3 of P0 gene. Autoantibodies were searched in patient's serum by a western blot analysis of human normal peripheral nerve extract. The peripheral nerve extract was a pool from sural nerve biopsy samples. Although we did not find antibodies against normal P0 protein in our case, we cannot exclude the presence of antibodies against mutated P0 protein.

A clinical examination of the proband's brother (43 years) and daughter (18 years) showed the presence of difficulties on gait on toes and heels, bilateral pes cavus, and areflexia. Nerve conduction studies revealed diffuse reduction in motor and sensory conduction. The mutation p.Val 102fs has been also identified in the proband's brother and daughter.

The patient was diagnosed with MS and CMT 1B disease and she was then started on treatment with glatiramer acetate.

In the following 2 years the patient develop one clinical relapse, whereas MRI remained stable compared with the previous one.

In the case presented here, clinical history, imaging, and cerebrospinal fluid findings supported the diagnosis of MS. In particular, conventional MRI showed the dissemination in space and in time typical of MS, and spectroscopy revealed abnormal lower NAA (indicating that neuronal damage had already occurred) and higher Cho (suggestive of active membrane turnover, most likely due to de‐ and remyelination; Belinda et al., 2003).

However, neurophysiological analysis and family history suggested the co‐occurrence of an autosomal inherited demyelinating neuropathy confirmed by the evidence of a point mutation in the P0 gene compatible with a diagnosis of CMT1B.

Few cases with a mutation in the MPZ gene and MS have been described until now and, to the best of our knowledge, this is the first case with a p.Val 102fs mutation (Table 1; Rajabally & Abbott, 2005). The p.Val102fs mutation in the MPZ gene has been reported previously, but no patient with this mutation showed a concomitant central nervous system involvement (Pareyson et al., 1999; Warner et al., 1996).

**Table 1:** Previously described cases of association between MS and CMT

References	No. of patients	Sex	Age of MS onset	Age of CMT onset	MS type	CMT type	MRI lesion's location	Mutation	Other diseases
Koutsis et al. (2015)	1	M	46	52	RRMS	CMTX	ST, IT, SC	Cx32	NM
Koros et al. (2013)	1	M	31	26	RRMS	CMT1A	ST, SC	PMP22	NM
Potulska‐Chromik et al. (2012)	1	F	25	20	PPMS	CMT1C	ST, SC	LITAF	NM
Parman et al. (2007)	1	M	20	16	RRMS	CMTX	ST, IT, SC	Cx32	NM
Kilfoyle et al. (2006)	1	F	27	37	NM	CMT1B	NM	P0	NM
Isoardo et al. (2005)	1	M	44	NM	PRMS	CMTX	ST, IT	Cx32	NM
Rajabally et al. (2005)	1	F	58	49	RRMS	NM	ST, IT, SC	P0	NM
Gil et al. (1999)	1	F	35	NM	NM	NM	ST	NM	Crohn's disease
Almsadi et al. (1998)	1	F	34	NM	NM	CMT1A	ST	PMP22	NM
Frasson et al. (1997)	2	2F	38; 22	NM; 30	NM	CMT1A	ST, SC	PMP22	NM
Mathews et al. (1972)	1	M	26	NM	NM	NM	NM	NM	Mixed glioma

Cx32, connexin 32; IT, infratentorial; LITAF, lipopolysaccharide‐induced tumor necrosis factor‐α; P0, protein zero; NM, not mentioned; PMP22, peripheral myelin protein 22; PPMS, primary progressive multiple sclerosis; PRMS, progressive relapsing multiple sclerosis; RRMS, relapsing remitting multiple sclerosis; SC, spinal cord; ST, supratentorial.

Given the very high prevalence of MS and the rarity of inflammatory and hereditary demyelinating neuropathy, the sporadic concomitant peripheral and central demyelination has been usually related to the chance (Potulska‐Chromik et al., 2012). However, a potential explanation of this association may be the presence of an autoimmune disorder involving both central and peripheral myelin (Kawamura et al., 2013; Potulska‐Chromik et al., 2012), only in a single case report of this association antifascin antibodies have been detected (Sharma et al., 2008). In the case presented here, we did not perform antifascin antibodies detection.

Data from literature reported the association between CMT (particularly CMT1B) and inflammatory neuropathy, supported by pathological studies on CMT1B animal models showing a lymphocytic infiltration with macrophages on the onion bulbs (Shy et al., 1997). The authors thereby hypothesized a proinflammatory potential role of mutated P0 protein involved in CMT1B pathogenesis due to molecular mimicry (Shy et al., 1997). Additionally, a genetic susceptibility to develop MS has been aimed in patients with CMT1C, in which mutations in LITAF gene would lead to a TNF‐α overexpression. A role of TNF‐α in the pathogenesis and outcome of MS has been widely demonstrated, thus supporting the hypothesis that a possible link between these conditions may be tied to a proinflammatory role of mutated myelin proteins involved in the pathogenesis of CMT diseases (Potulska‐Chromik et al., 2012).

Even if the association reported here is probably due to chance, the evidence of the increasing number of cases with CMT and MS may require larger prospective studies to clarify such a proinflammatory role.

## Conflict of Interest

Dr. Simone received honoraria from Genzyme Sanofi‐Aventis, TEVA, Bayer, and Biogen for educational lectures. Dr. Paolicelli received honoraria for consultancy and/or speaking from Biogen Idec, Merck Serono, Teva, Novartis, Almirall, Bayer Schering, and has served on scientific advisory boards for Biogen Idec and Merck Serono. No disclosures were reported by other authors.
